# Comparative transcriptome analysis of the human endocervix and ectocervix during the proliferative and secretory phases of the menstrual cycle

**DOI:** 10.1038/s41598-019-49647-3

**Published:** 2019-09-17

**Authors:** S. Mukhopadhyay, Y. Liang, H. Hur, G. Villegas, G. Calenda, A. Reis, L. Millen, P. Barnable, L. Mamkina, N. Kumar, T. Kalir, R. Sperling, N. Teleshova

**Affiliations:** 1 Population Council, New York, NY USA; 2 Bioinformatics Program, The Rockefeller University, New York, NY USA; 3 Icahn School of Medicine at Mt. Sinai, New York, NY USA

**Keywords:** Gene ontology, Mucosal immunology

## Abstract

Despite extensive studies suggesting increased susceptibility to HIV during the secretory phase of the menstrual cycle, the molecular mechanisms involved remain unclear. Our goal was to analyze transcriptomes of the endocervix and ectocervix during the proliferative and secretory phases using RNA sequencing to explore potential molecular signatures of susceptibility to HIV. We identified 202 differentially expressed genes (DEGs) between the proliferative and secretory phases of the cycle in the endocervix (adjusted *p < *0.05). The biofunctions and pathways analysis of DEGs revealed that cellular assembly and epithelial barrier function in the proliferative phase and inflammatory response/cellular movement in the secretory phase were among the top biofunctions and pathways. The gene set enrichment analysis of ranked DEGs (score = log fold change/*p* value) in the endocervix and ectocervix revealed that (i) unstimulated/not activated immune cells gene sets positively correlated with the proliferative phase and negatively correlated with the secretory phase in both tissues, (ii) IFNγ and IFNα response gene sets positively correlated with the proliferative phase in the ectocervix, (iii) HIV restrictive Wnt/β-catenin signaling pathway negatively correlated with the secretory phase in the endocervix. Our data show menstrual cycle phase-associated changes in both endocervix and ectocervix, which may modulate susceptibility to HIV.

## Introduction

Sex hormones control female reproductive tract (FRT) mucosal immune system and cyclical changes in hormone levels are implicated in the “window of vulnerability” theory, suggesting that HIV transmission is more likely to occur within 7–10 days after ovulation during the progesterone (P4) dominating secretory phase of the menstrual cycle rather than during the rest of the cycle^1–4^. This theory was supported by SHIV and SIV transmission studies in rhesus and pigtail macaques^5–10^. However, *in vitro* HIV challenge of human cervical tissues from subjects in the proliferative and secretory phases of the cycle demonstrated contrasting outcomes^11–13^. Our studies utilizing tissues collected over broad periods during the proliferative and secretory phases of the cycle showed similar HIV-1_BaL_ infection level in both phases of the cycle^13^. Tissue challenge close to ovulation at the peak of estradiol (E2) during the proliferative phase and at the peak of P4 concentrations during the secretory phase could have potentially revealed differences in tissue infection. Our results demonstrating an inverse association between serum E2 concentrations and endocervical and ectocervical tissue infection support this possibility and suggest E2-mediated protection against HIV acquisition^13^. Overall, these data underlie the need for studies to understand better how susceptibility to HIV is affected by the menstrual cycle in different mucosal areas of the FRT.

Ectocervical, endocervical and vaginal mucosa are distinct anatomical sites, all susceptible to HIV infection, as suggested by SIV transmission studies in rhesus macaques^14^, but with significant differences in HIV target T cell, macrophage and dendritic cell distributions^15,16^. Potential biomarkers influencing HIV transmission in the lower FRT have been explored by several groups. CD45^+^, CD3^+^, CD4^+^, CD8^+^, CCR5^+^, CXCR4^+^, HLA-DR^+^, CD1a^+^, CD68^+^ and GalCer^+^ populations in the ectocervix and/or vaginal tissue were found not to be affected by the menstrual cycle^12,15,17–19^. However, an increase in frequencies of CCR5^+^ CD4^+^ T cells in endocervical cytobrush specimens was reported in the luteal phase^20^.

Analysis of the transcriptome using microarrays demonstrated significant differences in endocervical^21^, but not in vaginal^12^, tissue gene expression between the phases of the cycle. The endocervical microarray data revealed genes associated with cell-matrix interactions, amino acid and lipid metabolism, and immune regulation in the follicular phase tissues. The luteal phase was primarily associated with genes involved in chromatin remodeling, inflammation, angiogenesis, oxidative stress and immune cell regulation^21^. To our knowledge, there is no published data on ectocervical gene expression changes during the menstrual cycle.

We aimed to analyze the ectocervical and endocervical tissue transcriptomes during the proliferative and secretory phases of the cycle using RNA sequencing (RNAseq) to explore potential signatures of susceptibility to HIV. Hysterectomy tissue specimens from subjects not using hormonal contraception/treatment for gynecological conditions were utilized. Several paired ecto- and endocervical specimens were included, minimizing intersubject variability. We chose RNAseq as a high-throughput means with low background signal and large dynamic range^22,23^.

Differentially expressed genes (DEGs) between proliferative and secretory phases of the cycle were identified in the endocervix and were subjected to the analysis of pathways, molecular networks and biofunctions^24^. To inquire into changes in gene expression irrespective of *p* value cut off, full ranked gene lists without any cutoff based on proliferative vs. secretory phase expression were subjected to gene set enrichment analysis (GSEA) against the Hallmark gene sets (H)^25^ and Immunologic Signatures gene sets (collection C7)^26^ from the Molecular Signatures Database (MSigDB). These analyses identified menstrual cycle phase-specific changes in both ectocervix and endocervix.

## Results

### Gene expression in the ecto- and endocervix and DEGs in the proliferative vs. secretory phases of the cycle

**Figure 1:**
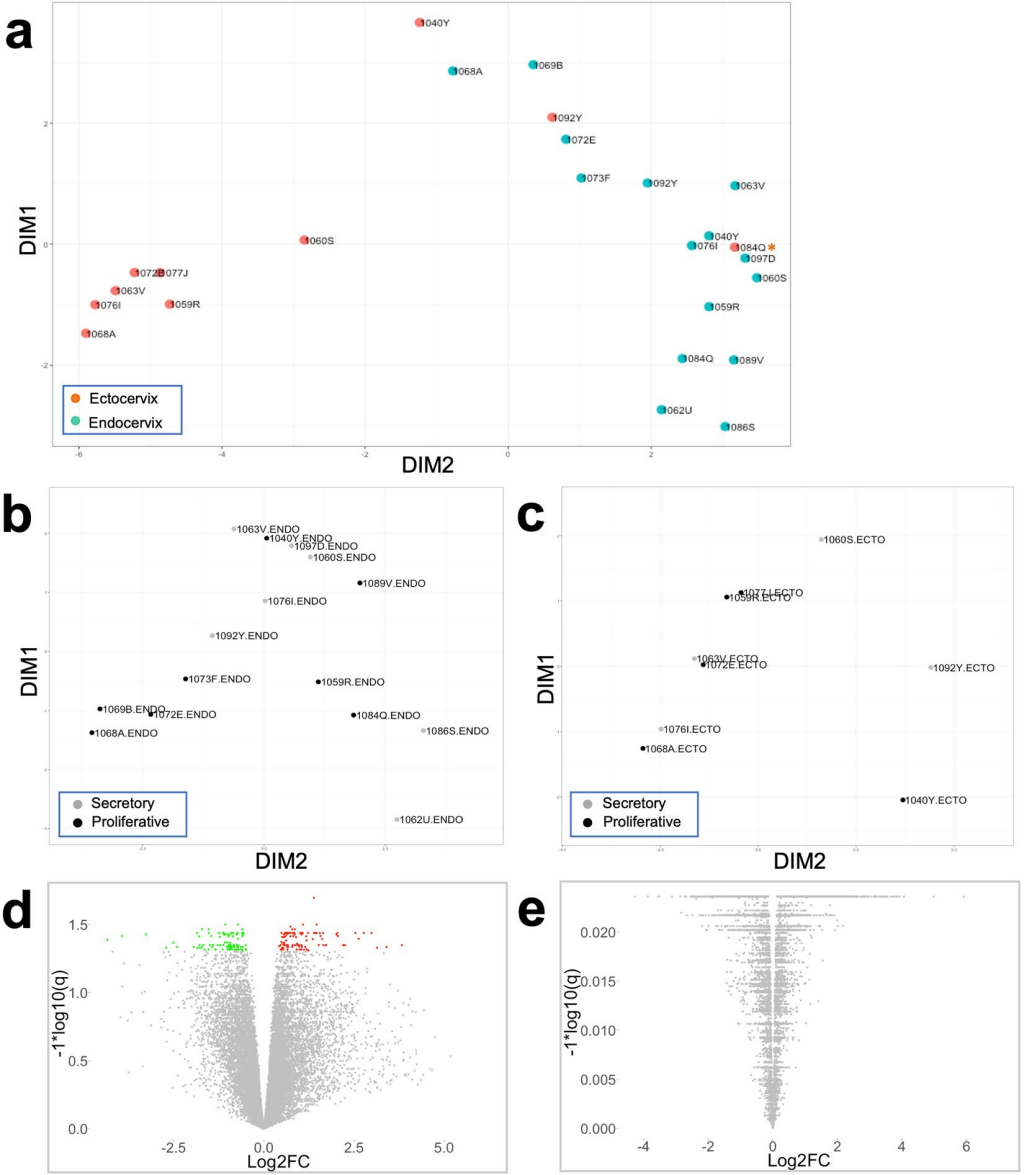
Ectocervical and endocervical tissue gene expression in the proliferative and secretory phases of the cycle. (**a)** MDS plot of endocervical and ectocervical gene expression. Top 500 genes were plotted based on expression differences (logFC) between the samples. 1084Q* ectocervical tissue sample clustered close to endocervical tissues and was excluded from subsequent analysis. **(b**,**c)** MDS plots of endocervical (**b**) and ectocervical (**c**) gene expression in the proliferative and secretory phases. **(d**,**e)** Volcano plots of endocervical (**d**) and ectocervical (**e**) gene expression in the proliferative vs. secretory phase of the cycle. In red are upregulated DEGs and in green are downregulated DEGs.

The study subjects were categorized into proliferative or secretory phase groups based on the histological assessment of the endometrium (Supplementary Table 1). Serum E2 and P4 concentrations were measured (Supplementary Table 1) and confirmed subjects’ categorization. To present data in unbiased way, an unsupervised analysis using multidimensional scaling (MDS) reduction of gene expression profiles in ectocervical (n = 10) and endocervical (n = 15) tissues was performed and is shown in Fig. 1a.

Ectocervix and endocervix samples tended to group separately on the MDS plot (Fig. 1a). Also, an intermediate cluster of six samples (1040Y ectocervix, 1068A endocervix, 1069B endocervix, 1092Y ectocervix, 1072E endocervix, 1073F endocervix) was noted. The paired ectocervical/endocervical samples from subjects in this cluster (1040Y, 1068A, 1092Y, 1072E) are located at a distance, pointing to the differences in gene expression between the two mucosal sites within an individual subject. Five of the six samples in the intermediate cluster were from subjects in the proliferative phase.

One ectocervical sample from a subject in the proliferative phase (1084Q) clustered with endocervical samples (Fig. 1a) and was excluded from the subsequent analysis. Based on routine pre-operative clinical cancer screening, this subject had positive HPV testing (Supplementary Table 1) and HPV infection was previously reported to be associated with altered gene expression in cervical epithelium^27,28^.

Phase-based gene expression profiles in the endocervix and ectocervix were mixed, with higher degree of separation in the endocervix (Fig. 1b,c), likely due to larger sample size in this group.

202 DEGs (adjusted *p* values < 0.05; 102 upregulated and 100 downregulated) were identified in the endocervix based on comparison of n = 8 vs. n = 7 samples from subjects in the proliferative and secretory phases, respectively (Supplementary Tables 2, 3). In the ectocervix, no DEGs were identified comparing n = 5 proliferative and n = 4 secretory phase samples. The volcano plots of gene expression in the proliferative vs. secretory phase according to the fold change and *p* values depict DEGs in the endocervix and lack of DEGs in the ectocervix (Fig. 1d,e).

### DEGs in the proliferative vs. secretory phases of the cycle in the endocervix

The upregulated DEGs in the proliferative vs. secretory endocervix ranked by fold change are listed the Supplementary Table 2. The complete data can be found in the NCBI’s Gene Expression Omnibus database (Accession Number:GSE122248)^29^. *FOSL1*, a regulator of cell proliferation and differentiation, which is induced by estrogen^30^, was the most upregulated gene in the proliferative endocervix. Consistent with regulatory role of estrogen in lipid metabolism^31,32^, several genes involved in lipid metabolism (*FADS1*, *SREBF2*, *PI4K2A*, *LDLR*)^33–36^ were upregulated. Expression of Cathepsin G was increased in accord with data demonstrating induction of serine proteases by estrogen^37^. Cyclin Y family member *CCNYL1* enhancing Wnt/β-catenin signaling in mitosis^38^, was also induced. Several genes involved in epithelial cell barrier function, microtubule cytoskeleton organization, cell division, microtubule structure/DNA stability, actin dynamics and cell motility, structure and intracellular signaling (*TUBB2A*, *TUBA1B*, *TUBB*, *TUBA1C*, *CFL1*, *CDC42*, Actin β (*ACTB*), *MYL6*)^39–43^ were upregulated.

The downregulated DEGs in the proliferative vs. secretory endocervix (accordingly representing upregulated genes in the secretory phase) ranked by fold change are listed in the Supplementary Table 3. The most downregulated gene in the proliferative phase was a serine proteinase inhibitor * SERPINA5*. Mucosal serpins were previously reported to be differentially regulated during the menstrual cycle, with some increasing in the proliferative phase (Serpin A1) and some (Serpin A3) remaining similar between the phases of the cycle^44^. The downregulated DEGs in the proliferative phase included genes associated with inflammatory pathways such as PLA2 group 6 gene (*PLA2G6*). *PLA2* promotes inflammation by catalyzing arachidonic acid pathway and regulates speed and directionality of monocytes’ chemotaxis^45^. *ENPP3*, a ectonucleotide pyrophosphatase/phosphodiesterase 3, which is expressed on basophils and mast cells^46^ and a marker of cell activation, was downregulated. *ENPP3* expression in uterus is suggested to be regulated by P4 and is increased during midsecretory phase when P4 is high^47^. *ADHFE1*, an enzyme mediating oxidative stress^48^, was downregulated. Also, a decrease in expression of broad range of nucleic acid binding zinc finger proteins was detected.

### Biological processes, pathways and molecular networks in the proliferative and secretory phase of the cycle in the endocervix

#### Biofunctions and pathways analysis using all DEGs

**Figure 2:**
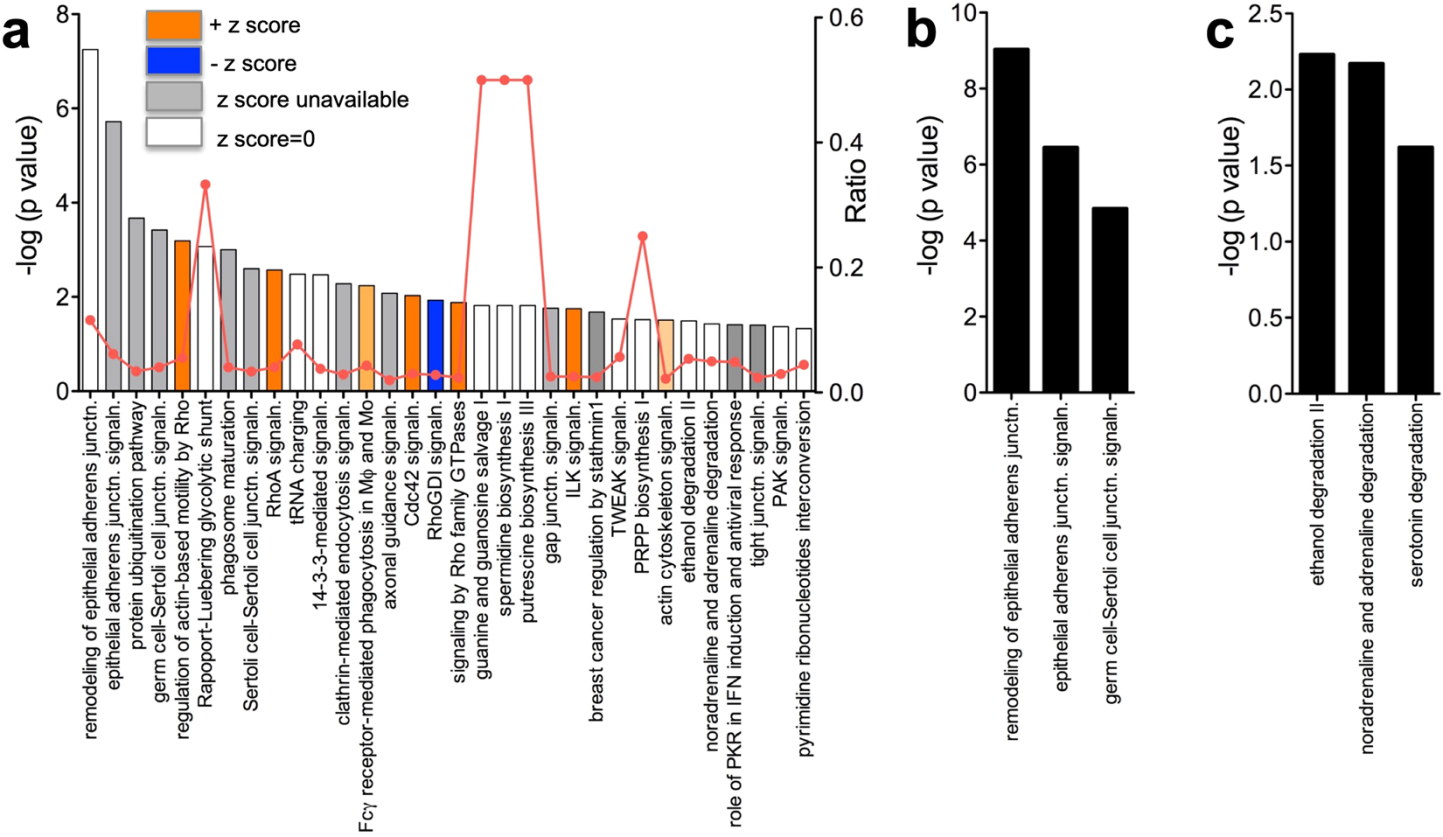
Canonical pathways associated with the proliferative or secretory phases of the cycle in endocervix. (**a)** Pathways significantly changed in the proliferative vs. secretory phases. The y-axis displays the −log (*p* value) which was calculated by right-tailed Fisher’s exact test. The default cut off -log (*p* value) of 1.3 was applied. (−) z scores indicate down-regulation in the proliferative endocervix and (+) z scores indicate upregulation of the pathways in the proliferative endocervix. Some z scores were unavailable or unpredicted as the eligibility criteria of z score algorithm were not met. Ratio denotes the number of significantly changed genes compared with the total number of genes within the pathway. **(b**,**c)** Pathways identified by ORA using upregulated sets of DEGs in the proliferative (**b**) and secretory (**c**) phases.

The analysis revealed that the top two molecular and cellular functions associated with the DEGs in the proliferative phase in the endocervix were (i) DNA replication, recombination and repair (*p* = 1.86E-03-5.83E-05) and (ii) cell death and survival (*p* = 7.66E-03-8.35E-05). The canonical pathway analysis revealed 32 pathways that were significantly changed between the phases of the cycle (Fig. 2a and Supplementary Table 4). Many of the pathways belonged to the categories representing cytoskeleton remodeling, cell and organ development.

In the proliferative endocervix, the analysis predicted activation state of pathways involving selected Rho family GTPases linked with multiple aspects of cellular regulation including the actin system, cellular morphology, migration, gene transcription, the cell cycle and phagocytosis^49^. Rho A and Cdc42 signaling was induced and the negative regulator of Rho family (RhoGDI) signaling was inhibited (Fig. 2a). Expectedly, regulation of actin-based motility by Rho and actin cytoskeleton signaling pathways, Fcγ receptor-mediated phagocytosis in macrophages and monocytes and ILK signaling were also activated. Several pathways were significantly different between the phases of the cycle, however, the z scores indicating activation state for these pathways were either unavailable or unpredicted as the eligibility criteria of z score algorithm were not met (Fig. 2a). These pathways were linked to epithelial barrier function (remodeling of epithelial adherens junctions, epithelial adherens junction signaling, gap junction and tight junction signaling), metabolism (guanine and guanosine salvage I, PRPP biosynthesis I, pyrimidine ribonucleotides interconversion, spermidine and putrescin synthesis, tRNA charging, Rapoport-Luebering glycolytic shunt, ethanol degradation II, non-adrenaline and adrenalin degradation), immune regulation (phagosome maturation, clathrin-mediated endocytosis, TWEAK signaling, role of PKR in IFN induction and antiviral response), cell cycle/DNA repair/cell growth (14-3-3-mediated signaling, TWEAK signaling, PAK signaling).

#### Pathway overrepresentation analysis (ORA) using overabundant gene sets

Next, we explored ORA^50^ using overabundant gene sets as an alternative strategy to identify pathways associated with the proliferative or secretory phases. In agreement with analysis of all DEGs, ORA identified pathways involved in epithelial barrier function (remodeling of epithelial adherens junctions (p = 9.3E-10), epithelial adherens junction signaling (p = 3.51E-07) associated with the proliferative phase (Fig. 2b). In the secretory phase, metabolic pathways were overrepresented (Fig. 2c).

#### Network and biofunctions analysis using overabundant gene sets

To explore significant biological functions and to determine non-directional relationships among the upregulated DEGs in the proliferative and secretory phases, the network and biofunctions analysis tool on overabundant gene sets was employed. Consistent with the analyses above, lipid, mineral and vitamin metabolism, cellular assembly and organization biofunctions were enhanced in the proliferative endocervix (Table 1). Cell cycle, cell signaling, inflammatory response and cellular movement were enhanced in the secretory endocervix (Table 1).

**Table 1:** Top networks, functions and subfunctions enriched in the proliferative and secretory phase in endocervix.

Molecules in network	Score	Focus molecules	Top diseases and functions	Subfunctions
**Proliferative phase**				
19S proteasome, 20 s proteasome, 26s Proteasome, Cg, ECEL1, FADS1, FOSL1, FSH, GARS, GLRX3, HISTONE, HPRT1, IGFBP4, Ikb, INSIG1, LDL, LDLR, Lh, NFkB (complex), ODC1, PI4K2A, PSA, PSMC3, PSMD, PSMD1, PSMD2, PSMD7, PSMD11, SREBF2, Srebp, STC2, TNFAIP6, TNFRSF12A, UBE2N, Ubiquitin	46	22	Lipid metabolism, small molecule biochemistry, vitamin and mineral metabolism	Synthesis, accumulation and homeostasis of steroid, sterol, lipid, phospholipid, triacylgycerol, vitamin E, IMP, GMP, spermine, spermidine, progesterone
ACTB, Actin, α catenin, α tubulin, AP2M, ARPC2, BCR (complex), β tubulin, CAPZB, CCT5, CDC42EP2, CDK4/6, CFL1, Ck2, CPM, Cr3, ERK1/2, FActin, Hsp90, LSP1, Mlc, MYL6, Myosin, NOP16, PDLIM4, PP2A, Rock, S100A4, S100A10, TUBA1B, TUBA1C, TUBB, TUBB2A, tubulin, tubulin (family)	35	18	Cellular assembly and organization, tissue development, cancer	Formation of cytoskeleton filaments, actin filaments, polymerization of filaments, plasma membrane formation filaments
ADRB, caspase, CD3, CTPS1, CYCS, cytochrome C, cytokine, DRAP1, ERK, FKBP1A, Gsk3, HDL, Histone h4, HMCN1, Hsp70, IgG, IKK, (complex), IL1, Immunoglobulin, Interferon α, Jnk, MED27, Mmp, MTORC1, NME1, PGAM1, Pkc(s), PPIA, RAN, RBM3, SLC25A5, SRM, TCR, YARS, YWHAQ	28	15	Dermatological diseases and conditions, organismal injury and abnormalities, nucleic acid metabolism	Biosynthesis of nucleoside triphosphate, exchange and synthesis of CDP, CTP, ADP, ATP
**Secretory phase**				
APBB3, APC, AVIL, BRIP1, C3orf62, C6orf163, FAM161A, AM214A, FAN1, FANCD2, FIGNL1, GOLGA2, ING5, INPPL1, JADE2, LYN, MEST, MINPP1, MTFR2, MYO1F, NAV2, NOP1, NUDT16, NUPR1, PCMTD2, PEX6, PPFIBP2, SLC25A29, TDRD6, TFB2M, TMCO6, UBB, ZBTB14, ZNF577, ZNF737	31	15	Cell cycle, connective tissue disorders, hereditary disorder	Cell cycle progression, ploidy, polyploidization
ADH6, ARRB1, BAZ2A, βestradiol, CCNG2, Q8A, ECHDC2, EGFR, ESR1, FARSA, GCSH, GNRH2, GTF2IRD1, HDGFL3, HEXDC, hexosaminidase, KIAA1107, KLHL24, KLK3, L-dopa, L3MBTL1, LRRC66, MTERF2, NPM1, PROC, PSD, RPS29, RSL1D1, SAMD11, YK, TESMIN, TMEM159, ZFP62, ZMYM3, Z NF137P	26	13	Cell signaling, molecular transport, small molecule biochemistry	Release of nitric oxide, concentration of corticosterone
5-oxo-6-8-11-14-(e,z,z,z)-eicosatetraenoic acid, ACVR2B, ADGRG5, ADP, α1 antitrypsin, ANKRA2, BIRC2, BMP3, C19orf44, CIRBP, Creb, ENPP3, ERK1/2, FN1, GLUD, HSF4, IGHE, Insulin, Jnk, KLKB1, leukotriene B4, MIA2, phosphatidylethanolamine, PLA2G6, PLAT, PPP1CA, PPP1R3E, PROC, prostaglandin D2, PTGDR2, SERPINA5, SIRT3, TCF, TSPAN10, ZNF546	23	12	Inflammatory response, cellular movement, hematological system development and function	Chemotaxis and recruitment of leucocytes (monocytes, T lymphocytes, basophils, eosinophils)

Score = Likelihood of focus molecules to be truly network eligible.

Focus molecules = Number of network eligible molecules per network.

### GSEA

To incorporate information on all genes expression without introducing *p* value cut off, endocervix and ectocervix limma outputs genes were ranked (score = log FC/p value) and then used as input to run against the Hallmark gene sets (H) (n = 50)^25^ and Immunologic Signatures gene sets (collection C7)(n = 4872)^26^ of the GSEA MSigDB. The top gene sets, which positively or negatively correlated with a particular phase of the cycle in the endo- and ectocervix are shown in Tables 2, 3 and Supplementary Figures 1, 2.

**Table 2:** Top positively and negatively correlated gene sets with the proliferative and secretory phase of the cycle (Hallmark database).

Gene set ID	Gene set description	NES	FDR q value	Size
**Proliferative endocervix**				
Hallmark-MYC-Targets-V1	A subgroup of genes regulated by MYC-version 1 (v1)	7.47	0.000	199
Hallmark-MTORC1-Signaling	Genes up-regulated through activation of mTORC1 complex	6.60	0.000	198
Hallmark-TNFα -Signaling-Via-NFKβ	Genes regulated by NF-kβ in response to TNF	6.44	0.000	195
Hallmark-Oxidative-Phosphorylation	Genes encoding proteins involved in oxidative phosphorylation	6.42	0.000	196
Hallmark-Epithelial-Mesenchymal-Transition	Genes defining epithelial-mesenchymal transition, as in wound healing, fibrosis and metastasis	5.99	0.000	196
**Secretory endocervix**				
Hallmark-WNT-β Catenin-Signaling	Genes up-regulated by activation of WNT signaling through accumulation of beta catenin CTNNB1	−0.97	0.938	40
Hallmark-Bile-Acid-Metabolism	Genes involved in metabolism of Bile and salts	−0.81	0.699	96
**Proliferative ectocervix**				
Hallmark-Interferon γ Response	Genes up-regulated in response to IFNγ	4.27	0.000	192
Hallmark-MYC-Targets-V1	A subgroup of genes regulated by MYC-version 1 (v1)	4.10	0.000	199
Hallmark-G2M-Checkpoint	Genes involved in the G2/M checkpoint, as in progression through the cell division cycle	3.99	0.000	196
Hallmark-E2F-Target	Genes encoding cell cycle related targets of E2F transcription factors	3.88	0.000	199
Hallmark-Interferon α Response	Genes up-regulated in response to IFNα proteins	3.52	0.000	92
**Secretory ectocervix**				
Hallmark-UV-Response-DN	Genes down-regulated in response to ultraviolet (UV) radiation	−2.54	0.001	142
Hallmark-Epithelial-Mesenchymal-Transition	Genes defining epithelial-mesenchymal transition, as in wound healing, fibrosis and metastasis	−2.27	0.007	188
Hallmark-Protein-Secretion	Genes involved in protein secretion pathway	−2.12	0.012	95
Hallmark-KRAS-Signaling-Up	Genes up-regulated by KRAS activation	−1.69	0.087	178
Hallmark-Angiogenesis	Genes up-regulated during formation of blood vessels (angiogenesis)	−1.50	0.166	30

NES = Normalized enrichment score.

FDR q value = FDR adjusted p value. Estimated probability that the NES represents a false finding.

Size = Number of genes in the gene set after filtering the genes not present in expression data. + NES indicates positive correlation with a particular phase.

−NES indicates negative correlation with a particular phase.

**Table 3:** Top positively and negatively correlated gene sets with the proliferative and secretory phase of the cycle (Immune database).

Gene set ID	Gene set description	NES	FDR- q value	Size
**Proliferative endocervix**				
GSE22886	Genes down-regulated in comparison of unstimulated NK cells versus those stimulated with IL15 [Gene ID = 3600] at 16 h.	7.12	0.000	194
GSE29618	Genes down-regulated in comparison of B cells versus myeloid dendritic cells (mDC).	7.01	0.000	196
GSE3982	Genes down-regulated in comparison of memory CD4 [GeneID = 920] T cells versus Th1 cells.	6.83	0.000	189
GSE2405_0H (24 hrs)	Genes up-regulated in polymorphonuclear leukocytes (24 h): control versus infection by A. phagocytophilum.	6.82	0.000	192
GSE2405_0H (9 hrs)	Genes up-regulated in polymorphonuclear leukocytes (9h): control versus infection by A. phagocytophilum	6.74	0.000	192
**Secretory endocervix**				
GSE2706	Genes up-regulated in comparison of unstimulated dendritic cells (DC) at 0 h versus DCs stimulated with LPS (TLR4 agonist) for 2 h	−4.43	0.000	155
GSE2706	Genes up-regulated in comparison of unstimulated dendritic cells (DC) at 0 h versus DCs stimulated with LPS (TLR4 agonist) and R848 for 2 h.	−4.34	0.000	164
GSE2405	Genes down-regulated in polymorphonuclear leukocytes 9 h after infection by: S. aureus versus A. phagocytophilum.	−3.52	0.000	190
GSE18791	Genes up-regulated in comparison of control conventional dendritic cells (cDC) at 0 h versus cDCs infected with Newcastle disease virus (NDV) at 2 h.	−3.52	0.000	161
GSE22886	Genes up-regulated in comparison of unstimulated NK cells versus those stimulated with IL2 at 16 h.	−3.27	0.000	176
**Proliferative ectocervix**				
GSE9006	Genes up-regulated in comparison of peripheral blood mononuclear cells (PBMC) from healthy donors versus PBMCs from patients with type 2 diabetes at the time of diagnosis.	5.83	0.000	188
GSE9006	Genes up-regulated in peripheral blood mononuclear cells (PBMC) from patients with type 1 diabetes at the time of diagnosis versus those with type 2 diabetes at the time of diagnosis.	5.65	0.000	190
GSE36476	Genes down-regulated in comparison of untreated CD4 memory T cells from old donors versus those treated with TSST at 40 h.	5.38	0.000	190
GSE36476	Genes down-regulated in comparison of untreated CD4 memory T cells from young donors versus those treated with TSST at 40 h.	5.23	0.000	190
GSE16450	Genes down-regulated in the neuron cell line: immature versus mature.	5.18	0.000	173
**Secretory ectocervix**				
GSE21774	Genes downregulated in CD56 ^Bright^ CD62L^±^ NK cells versus CD56 ^Dim^ CD62L^−^ NK cells	−3.60	0.000	183
GSE14000	Genes up-regulated in comparison of polysome bound (translated) mRNA versus total mRNA 4 h after LPS (TLR4 agonist) stimulation.	−3.52	0.000	183
GSE26928	Genes down-regulated in comparison of CD4 [GeneID = 920] effector memory T cells versus CD4 [GeneID = 920] central memory T cells.	−3.50	0.000	131
GSE18791	Genes up-regulated in comparison of control conventional dendritic cells (cDC) at 0 h versus cDCs infected with Newcastle disease virus (NDV) at 2 h	−3.36	0.000	159
GSE13738	Genes up-regulated in comparison of resting CD4 [GeneID = 920] T cells versus directly activated CD4 [GeneID = 920] T cells	−3.21	0.001	164

NES = Normalized enrichment score.

FDR q value = FDR adjusted p value. Estimated probability that the NES represents a false finding.

Size = Number of genes in the gene set after filtering the genes not present in expression data. + NES indicates positive correlation with a particular phase.

−NES indicates negative correlation with a particular phase.

The Hallmark database analysis detected positive correlation of growth and proliferation related gene sets (mTOR1 signaling, oxidative phosphorylation, MYC targets V1 and epithelial-mesenchymal transition)^51–54^ and TNFα signaling with the proliferative phase in the endocervix. In the secretory endocervix, negative correlation with Wnt/β-catenin signaling, was revealed. Similar to the results in the endocervix, cell growth related gene sets (MYC Targets V1, G2M Checkpoint and E2F target)^55–58^ positively correlated with proliferative phase in the ectocervix. Also, a positive correlation between gene sets induced in response to IFNγ and IFNα and the proliferative phase in ectocervix was noted. In contrast, negative correlation between several cell proliferation-related gene sets (KRAS Signaling, angiogenesis and epithelial-mesenchymal transition)^59–61^ and the secretory phase in the ectocervix was detected.

The Immunologic Signatures  database analysis revealed positive correlations between (i) unstimulated NK cells, uninfected/resting neutrophils gene sets and the proliferative phase in the endocervix, (ii) resting/untreated CD4 T cells gene sets and the proliferative phase in ectocervix. Negative correlations were observed between (i) unstimulated immune cells (DCs, NK cells) gene sets and the secretory phase in endocervix, and (ii) unstimulated/untreated DCs and CD4 T cells, CD56^+bright^CD62L^+^ NK cells and the secretory phase in the ectocervix.

## Discussion

To our knowledge this is the first report to explore ectocervical and endocervical tissue transcriptomes relative to the menstrual cycle phases using RNAseq. The conducted analyses of DEGs in the endocervix and full ranked data analysis in the endocervix and ectocervix revealed complementary hypothesis-generating information on how susceptibility to HIV may be regulated during the menstrual cycle.

The unbiased analysis of gene expression pattern between ectocervix and endocervix and within each tissue during the proliferative and secretory phases of the cycle showed variable separation between the groups. RNAseq capability to detect weakly expressed genes more efficiently than other platforms^62,63^ and small sample size could have contributed to the observed gene expression pattern on the MDS plots. Studies involving larger sample size will help characterize better tissue-associated and phase-associated gene expression patterns in the ectocervix and endocervix.

The functional annotation of DEGs identified between the proliferative and secretory phase of the cycle in the endocervix is largely in agreement with findings by microarray^21^. However, RNAseq identified more DEGs than reported by the microarray (n = 110). Based on the manual comparison, only six DEGs (*ECHDC2*, *PNPLA7*, *DRAP1*, *FKBP1A*, *IGFBP4*, *ACTB*) overlapped between the studies. Similar with the microarray data, we did not see any of the mucin genes among the DEGs.

Among identified DEGs, literature review revealed several genes influencing HIV pathogenesis through protein-protein interactions. This includes genes which regulate HIV infectivity (*EHD4*, *AP2M1*)^64–66^, entry/assembly/budding (*INSIG1*, *PI4K2A*)^67,68^, proteasomal degradation (*LSP1*)^69^, HIV transcription/latency (*TFAP4*, *MED27*, *FOSL1*, *PSMC3*, *PSMD11*, *L3MBTL1*)^70–75^, viral transport (*ALYREF*, *MAPRE1* (*EB1)*)^76,77^, and actin dynamic involved in multiple steps during HIV life cycle (*CFL1*)^78,79^. Majority of these genes were upregulated and *TFAP4*, *L3MBTL1* were downregulated in the proliferative phase. No phase-associated HIV enhancing or inhibitory pattern of these genes was evident.

In the proliferative endocervix, pathways involved in barrier function maintenance (remodeling of epithelial adherens junctions, epithelial adherence junction signaling) were overrepresented possibly indicating effective epithelial barrier, that can mediate protection against HIV acquisition^80^. Several pathways cascaded to activation of cytoskeleton development (Rho A and actin cytoskeleton signaling) and immune responses (Fcγ receptor-mediated phagocytosis in macrophages and monocytes, ILK signaling). Growth and proliferation-related gene sets, TNFα signaling, unstimulated immune cells gene sets (neutrophils, NK cells) positively correlated with the proliferative phase. Unstimulated immune cells gene signatures point to HIV non-permissive environment. Resting neutrophils (vs. activated) bind HIV less effectively and are less efficient at HIV transfer to lymphocytes^81^. Presence of resting NK cells is an indicator of non-inflammatory environment in the mucosa during the proliferative phase, as NK cells are activated by proinflammatory cytokines^82^. Downregulation of *SERPINA5* in the proliferative phase deserves further exploration as a balance between serine proteases and their inhibitors was suggested to influence susceptibility to HIV^83^.

In the secretory endocervix, enhanced expression of genes related to inflammation, oxidative stress, and immune cell migration, potentially contributing to increased susceptibility to HIV^84,85^, was evident. Unstimulated/resting DCs and NK cells gene sets negatively correlated with the secretory phase. Furthermore, the E2-induced restrictive for HIV replication Wnt/β-catenin signaling pathway^86^ gene set also negatively correlated with the secretory phase.

The analysis of the ectocervix did not reveal DEGs between the follicular and secretory phases of the cycle. These data concur with the microarray results obtained in the vaginal mucosa, showing no difference in vaginal gene expression (greater than two fold) between the follicular and luteal phases^12^ with an exception of one gene (*HAL)*, which was significantly upregulated in the luteal phase. Similar to data in the proliferative endocervix, the full ranked data analysis revealed positive correlation between cell growth-associated gene sets, not-activated CD4 T cells gene sets and proliferative ectocervix. IFNα and IFNγ induced gene sets also positively correlated with the proliferative phase in ectocervix. Vaginal administration of Type I IFN (IFNβ) was shown to prevent SHIV infection in macaques^87^ and blockade of IFN-I receptor led to reduced anti-viral gene expression, increased SIV reservoir size and accelerated CD4 depletion with progression to AIDS^88^. However, it needs to be noted that the role of IFNs in HIV transmission and pathogenesis is complex^89,90^.

The secretory phase of the cycle in ectocervix negatively correlated with unstimulated DCs; resting CD4 T cells, which are less susceptible to HIV than activated CD4 T cells^91^; and CD56^+bright^CD62L^+^ NK cells, which polyfunctionality potentially relates to resistance to HIV-1 infection^92^. These data are indicative of higher susceptibility to HIV during the secretory phase in the ectocervix.

It needs to be acknowledged that although next generation sequencing technologies have advanced sequence-based research with the advantages of high-throughput, high-sensitivity, and high-speed, RNAseq analysis harbors limitations^22,23^. Messenger RNA concentrations do not necessarily reflect protein concentrations, which are influenced by transcription and translation rates and protein half-life. However, RNAseq transcriptome data can be used as a template for generation of proteomics database^93^.

Our study corroborates and expands the microarray data in the endocervix^21^ and is concurrent with cervico-vaginal lavage fluids proteome data, demonstrating dominance of pathways associated with inflammation, including chemotaxis and recruitment of leucocytes in the secretory phase^94^. The study offers insights into how susceptibility to HIV may be regulated during the menstrual cycle in the endocervix and ectocervix. Overall, competent epithelial barrier function, cytoskeletal organization, phagocytosis in monocytes/macrophages, immune responses, lack of inflammation and cellular activation (NK cells, neutrophils) may render endocervix resistant to infection with HIV during the proliferative phase. In contrast, in the secretory endocervix, compromised epithelial barrier function, inflammation, influx of leucocytes, cellular activation (NK cells, DCs), oxidative stress and decreased HIV-restrictive Wnt/β-catenin signaling may lead to enhanced susceptibility to HIV. Similarly, in the ectocervix, lack of cellular activation (T cells) and IFN γ and IFNα-induced responses potentially contribute to protection against HIV during the proliferative phase and cellular activation (T cells, DC), loss of CD56^+bright^ CD62L^+^ NK cells may lead to HIV permissive environment in the secretory phase.

Our study revealed gene expression signatures characteristic of menstrual cycle phase in the endocervical and ectocervical mucosa. This data may contribute to better understanding of susceptibility to mucosal HIV infection and other sexually transmitted infections as well as to development of prevention strategies.

## Materials and Methods

### Subjects

The project was approved by the Icahn School of Medicine at Mount Sinai Program for the Protection of Human Subjects (protocol #11-01380) and The Population Council IRB. The research was performed in accordance with relevant guidelines and regulations. Cervical tissues, blood and urine were obtained from women undergoing hysterectomies for non-malignant conditions (menometrorrhagia, leiomyomas, chronic pelvic pain, and pelvic organ prolapse) at Mount Sinai Hospital, the primary teaching hospital of the medical school. Subjects were enrolled after providing written informed consent. This is a sub-analysis from the 16 subjects who did not use either (i) hormonal contraception and/or (ii) any hormonal treatments for gynecological conditions within the 3 months prior to surgery. Age, race, phase of the cycle, histopathology report [cervical inflammation, metaplasia (for endocervical mucosa), parakeratosis (for ectocervical mucosa)], HSV-2 status and Pap cytology and HPV co-testing results of subjects included in individual data sets are summarized in Supplementary Table 1. The cycle phase was determined by assessing the histopathology of hematoxylin-and-eosin stained sections of the endometrial mucosa by board-certified diagnostic gynecologic pathologists who perform this assessment routinely. Cervical inflammation was defined as the presence of white blood cells within the cervical tissue (epithelium and stroma of the mucosa).

### Surgical tissue collection

Cervical tissues not used for clinical diagnosis which would otherwise be discarded were collected and released by pathologists within 1-2 hours (hrs) of surgery. Endocervix and ectocervix, excluding transformation zone, were utilized. Tissue samples were transported to the laboratory in RPMI on ice and a portion of mucosa (~3–5 mm) was placed in RNA*later* (Ambion) and then stored at −80 °C before RNA isolation.

### Radioimmunoassay (RIA)

Serum was kept at −80 °C before RIA. Hormone concentrations were determined using ImmuChem Double antibody 125I RIA kits for E2 (LLOQ 10 pg/ml) and for P4 (LLOQ 200 pg/ml) (MP Biomedicals, LLC, NY, USA).

### HSV-2 IgG AB Herpeselect test

Serum was kept at −80 °C before being assayed by Quest Diagnostics.

### Total RNA Extraction, Library Preparation & RNA Sequencing

Total RNA was isolated from tissues frozen in RNA*later* (Ambion) following manufacturer’s instructions (Qiagen RNeasy Fibrous Tissue Mini Kit). After extraction, the quality and the purity of the RNA were measured by the Agilent Bioanalyzer (Agilent, Santa Clara, CA). RNA was labeled and sequenced at the Rockefeller University Genomics center by using Illumina TruSeq technology (75 bp, > 30 M coverage). The FASTQ files were first quality controlled through FastQC v0.11.15^95^ with default parameters. Cutadapt v1.9.1 was used to locate and remove the adapter sequences from each high-throughput sequencing reads before mapping^96^ as applied to trim the low quality bases and TrueSeq adapters (–times = 2–quality-base = 33 —quality-cutoff = 5–format = FASQT–minimum-length = 25 -a AAAAAAAAAAAAA TTTTTTTTTTTTT -a AGATCGGAAGAG -a CTCTTCCGATCT). Trimmed FASQT files were aligned to the human genome (GRCH37) using STAR v2.4.2^97^ aligner with default parameters. The alignment results were then evaluated through Qualimap v.2.2 (10.1093/bioinformatics/bts503)^98^ to ensure that all the samples had a consistent coverage, alignment rate, and no obvious 5′ or 3′ bias. Aligned reads were then summarized through feature Counts v1.5^99^. The gene model used for this purpose was that from Ensembl at gene level. The uniquely mapped reads (NH ‘tag’ in bam file) that overlapped with an exon (feature) by at least 1 bp were counted and then the counts of all exons annotated to an Ensembl gene (meta features) and were summed into a single number. edgeR* v 3.16.5^100^ was used to normalize the samples and Voom from limma# v 3.30.11 was applied to estimate the differential log fold change in the expression of genes. An adjusted *p* value of less than 0.05 (*p* adj. < 0.05) was used to shortlist the genes that have a significant expression change.

### RNAseq data accession number

Raw sequence reads were uploaded to the Gene Expression Omnibus (GEO) Database (GEO Series Accession Number: GSE122248).

### Analysis of DEGs

#### MDS plots

MDS plots were prepared through plot MDS function within edgeR (10.1093/bioinformatics/btp616), which is a widely accepted RNAseq analytical package. It plots samples on a two-dimensional scatterplot so that distances on the plot approximate the expression differences (logFC) between the samples. Top 500 genes were plotted, which is the default value from the package.

#### Ingenuity pathway analysis (IPA)

To identify relevant cellular pathways differentially expressed between the proliferative and secretory phases of the cycle, list of DEGs containing gene identifiers, corresponding *p* values and log2 FC were subjected to the the IPA^24^ core analysis (Ingenuity Systems®) using default settings and uploading the DEGs, corresponding fold change expression values and FDR adjusted *p* values (aka *q* values)(<0.05). The analysis derives its results from the Ingenuity Knowledge Database, which is manually curated from published data. The canonical pathway component of the core analysis converted gene expression data into pathway illustrations and identified the top canonical pathways associated with the genes differentially expressed between two phases. −(*log*) *p* -value were calculated using the right-tailed Fisher’s Exact Test. A cut-off of 1.3 was applied to display only significantly changed pathways.

For ORA, we performed separate IPA core analysis with upregulated DEG datasets in each phase. Top gene networks associated with each phase were generated by default algorithm, which randomly selects focus genes from the uploaded list and draws connections on the basis of biological function from its knowledge database. The analysis also indicates the relevance of molecules to its assigned network.

### GSEA

Rank list file was created from limma output after proliferative vs. secretory phase gene expression comparison in the endocervix and ectocervix with each gene ranked by logFC/*p* value. Then the rank lists were used as an input of GSEA and analyzed through “Run GSEA on a Pre-Ranked” gene list option against gene sets databases (Hallmark gene sets^25^ and  Immunologic Signatures gene sets^26^).

## Supplementary information

Supplementary tables and figures
