# Supplementary material for: Comparative transcriptome analysis of the human endocervix and ectocervix during the proliferative and secretory phases of the menstrual cycle
Source: Sci Rep. 2019 Sep 17;9:13494. doi: 10.1038/s41598-019-49647-3 (PMC6749057; doi:10.1038/s41598-019-49647-3)
Supplement: Supplementary file 1 — Supplementary tables and figures [file 41598_2019_49647_MOESM1_ESM.pdf]

Comparative transcriptome analysis of the human endocervix and ectocervix during the proliferative and secretory phases of the menstrual cycle

S. Mukhopadhyay[1^], Y. Liang[2^], H. Hur[2], G. Villegas[1], G. Calenda[1], A. Reis[3], L. Millen[3], P. Barnable[1], L. Mamkina[1], N. Kumar[1], T. Kalir[3], R. Sperling[3], N. Teleshova[1*]

[1]Population Council, New York, NY, USA

[2]Bioinformatics Program, The Rockefeller University, New York, NY, USA

[3]Icahn School of Medicine at Mt. Sinai, New York, NY, USA

[^]Authors contributed equally

*Correspondence to nteleshova@popcouncil.org

Supplementary Table 1. Study cohort, tissue samples characteristics and serum hormonal concentrations

Patient ID	Race^	Age	Endo-metrial histology	Para-kera-tosis	Meta-plasia	Cervical inflam-mation	HSV-2 status	Last Pap test results*	History of abnormal Pap	Endo-cervix	Ecto-cervix	E2 (pg/ml)	P4 (pg/ml)
1040Y	White	44	P	-	+	-	-	normal	No	✓	✓	104.790	ND
1059R	White	48	P	-	-	+	+	normal	No	✓	✓	109.120	ND
1068A	White	42	P	+	-	-	-	normal	No	✓	✓	599.02	ND
1069B	White	50	P	-	+	-	-	normal	No	✓	-	148.990	ND
1072E	White	53	Weekly P	-	+	-	+	normal	Yes	✓	✓	120.290	ND
1073F	White	36	P	+	-	-	-	normal	Yes	✓	-	51.420	ND
1077J	Black	35	P	+	-	-	-	normal	No	-	✓	61.010	ND
1084Q	Black	47	P	-	-	-	+	HPV+	Yes	✓	✓	166.980	370.0
1089V	White	46	P	+	-	-	-	normal	No	✓	-	126.330	460.0
1060S	Black	44	S	-	-	-	-	normal	No	✓	✓	152.740	4100.0
1062U	White	45	S	-	-	-	-	normal	Yes	✓	-	174.530	7880.0
1063V	Black	50	S	+	-	-	-	normal	No	✓	✓	461.070	3180.0
1076I	White	43	Early S	-	-	+	-	normal	No	✓	✓	276.990	2970.0
1086S	Black	40	S	-	-	-	+	normal	No	✓	-	449.670	22580.0
1092Y	Black	43	Late S	+	-	-	-	normal	No	✓	✓	134.300	3620.0
1097D	White	41	S	+	-	-	+	normal	No	✓	-	88.810	12040

^Hispanic and Non-Hispanic ethnicity
* Pap test within ~1 year before surgery
P - proliferative phase
S - secretory phase
ND - not detected
E2 LLOQ=10 pg/ml
P4 LLOQ=200 pg/ml

Supplementary Table 2. Significantly upregulated endocervical genes in the follicular vs. secretory phase

Symbol	Entrez Gene Name	logFC	Adj.*p* value
FOSL1	FOS like 1, AP-1 transcription factor subunit	3.830417153	0.044919395
ECEL1	Endothelin converting enzyme like 1	3.403847637	0.046287061
RP11-334E6.12	RNA gene	3.138141528	0.046954048
TNFAIP6	TNF alpha induced protein 6	2.98402697	0.03660934
GPR3	G protein-coupled receptor 3	2.771447301	0.03660934
C5AR2	complement component 5a receptor 2	2.630776012	0.040375139
TNFRSF12A	TNF receptor superfamily member 12A	2.538815608	0.03660934
CTSG	Cathepsin G	2.252340776	0.044919395
HBEGF	Heparin binding EGF like growth factor	2.203869134	0.044919395
LDLR	Low density lipoprotein receptor	2.05247274	0.038511708
THY1	Thy-1 cell surface antigen	2.037749947	0.03660934
STC2	Stanniocalcin 2	2.013302887	0.037507157
TUBB2A	Tubulin beta 2A class IIa	1.730672336	0.044919395
ZNF367	Zinc finger protein 367	1.72394747	0.044919395
CPM	Carboxypeptidase M	1.651727396	0.044919395
LTBP1	Latent transforming growth factor beta binding protein 1	1.641118154	0.03660934
IGFBP4	Insulin like growth factor binding protein 4	1.61691717	0.040554146
TUBA1C	Tubulin alpha 1c	1.571507243	0.038511708
S100A10	S100 calcium binding protein A10	1.552591853	0.03660934
LSP1	Lymphocyte-specific protein 1	1.520932542	0.044772915
PRSS23	Protease, serine 23	1.468042873	0.03660934
INSIG1	Insulin induced gene 1	1.464397793	0.031708253
PDLIM4	PDZ and LIM domain 4	1.428033622	0.03660934
CCND1	Cyclin D1	1.390804817	0.03660934
TUBA1B	Tubulin alpha 1b	1.385038693	0.020200444
HMCN1	Hemicentin 1	1.35596791	0.040554146
CTPS1	CTP synthase 1	1.231992238	0.049108032
FAM222A	Family with sequence similarity 222 member A	1.22251027	0.03660934
ACTB	Actin beta	1.183945907	0.041522252
FADS1	Fatty acid desaturase 1	1.183422649	0.048251772
ODC1	Ornithine decarboxylase 1	1.170157266	0.038708533
S100A4	S100 calcium binding protein A4	1.1496318	0.046287061
CDC42EP2	CDC42 effector protein 2	1.111387395	0.04835945
RBM3	RNA binding motif (RNP1, RRM) protein 3	1.080035994	0.031708253
SRM	Spermidine synthase	1.078501824	0.03660934
NME1	NME/NM23 nucleoside diphosphate kinase 1	1.050073373	0.046287061
CYCS	Cytochrome c, somatic	1.043069525	0.037507157
EHD4	EH domain containing 4	1.042865018	0.044919395
TIGAR	TP53 induced glycolysis regulatory phosphatase	1.037051802	0.048251772
TUBB	Tubulin beta class I	1.036690502	0.03660934
MYL6	Myosin light chain 6	1.035661524	0.03660934
SLC25A5	Solute carrier family 25 member 5	1.002888338	0.03660934
KCNK6	Potassium two pore domain channel subfamily K member 6	0.983133172	0.04835945
CCNYL1	Cyclin Y like 1	0.97885213	0.045931911
NOP16	NOP16 nucleolar protein	0.978742415	0.044919395
TAF13	TATA-box binding protein associated factor 13	0.927677252	0.044919395
PGAM1	Phosphoglycerate mutase 1	0.920039989	0.043764693
CFL1	Cofilin 1	0.906552828	0.048251772
SH3KBP1	SH3 domain containing kinase binding protein 1	0.890563418	0.039150402

ANAPC15	Anaphase promoting complex subunit 15	0.862158508	0.044919395
DRAP1	DR1 associated protein 1	0.857704808	0.034319826
AEN	Apoptosis enhancing nuclease	0.854992908	0.037507157
BYSL	Bystin like	0.842702623	0.03660934
ETV5	ETS variant 5	0.839810085	0.03660934
MAPRE1	Microtubule associated protein RP/EB family member 1	0.82183947	0.03660934
C11orf24	Chromosome 11 open reading frame 24	0.809545381	0.038511708
ARPC2	Actin related protein 2/3 complex subunit 2	0.797095642	0.044919395
CAPZB	Capping actin protein of muscle Z-line beta subunit	0.794084722	0.03660934
ALYREF	Aly/REF export factor	0.781338061	0.034367731
CAPN5	Calpain 5	0.77853256	0.046287061
RAN	RAN, member RAS oncogene family	0.739692207	0.032817658
FKBP1A	FK506 binding protein 1A	0.7354102	0.038708533
PNO1	Partner of NOB1 homolog	0.730545389	0.03660934
SNRPD1	Small nuclear ribonucleoprotein D1 polypeptide	0.729432544	0.03660934
PPIA	Peptidylprolyl isomerase A	0.696775754	0.03660934
BZW2	Basic leucine zipper and W2 domains 2	0.682284085	0.03660934
LRRFIP2	LRR binding FLII interacting protein 2	0.671953412	0.046287061
PSMD1	Proteasome 26S subunit, non-ATPase 1	0.662554412	0.03660934
PI4K2A	Phosphatidylinositol 4-kinase type 2 alpha	0.662148135	0.044919395
PSMA7	Proteasome subunit alpha 7	0.661131248	0.03660934
MED27	Mediator complex subunit 27	0.657746272	0.044919395
EIF2S1	Eukaryotic translation initiation factor 2 subunit alpha	0.652196348	0.04835945
YKT6	YKT6 v-SNARE homolog	0.648237695	0.046287061
NOL6	Nucleolar protein 6	0.635380513	0.036756752
HPRT1	Hypoxanthine phosphoribosyltransferase 1	0.631259333	0.044919395
UBE2N	Ubiquitin conjugating enzyme E2 N	0.630669494	0.037507157
GARS	Glycyl-tRNA synthetase	0.620233757	0.03660934
PSMD7	Proteasome 26S subunit, non-ATPase 7	0.616048907	0.041556634
YARS	Tyrosyl-tRNA synthetase	0.611055715	0.03660934
GHITM	Growth hormone inducible transmembrane protein	0.596743289	0.038708533
GLRX3	Glutaredoxin 3	0.587786523	0.03660934
MRTO4	MRT4 homolog, ribosome maturation factor	0.587586966	0.03660934
PSMC3	Proteasome 26S subunit, ATPase 3	0.572398783	0.044919395
DCUN1D5	Defective in cullin neddylation 1 domain containing 5	0.566625127	0.044919395
ELOC	Elongin C	0.562982783	0.043033761
UTP11	UTP11, small subunit processome component homolog	0.562785212	0.044555005
YWHAQ	Tyrosine 3-monooxygenase/tryptophan 5-monooxygenase activation protein theta	0.560159671	0.044555005
SNRPF	Small nuclear ribonucleoprotein polypeptide F	0.557244417	0.03917199
ENSA	Endosulfine alpha	0.5538429	0.037507157
ZPR1	ZPR1 zinc finger	0.535455931	0.046954048
CMSS1	Cms1 ribosomal small subunit homolog (yeast)	0.534162426	0.048251772
PSMD11	Proteasome 26S subunit, non-ATPase 11	0.524281388	0.043853578
NUP93	Nucleoporin 93	0.51940754	0.04835945
CCT5	Chaperonin containing TCP1 subunit 5	0.510826992	0.048251772
SREBF2	Sterol regulatory element binding transcription factor 2	0.505582554	0.046954048
CDC42	Cell division cycle 42	0.505465545	0.039150402
CHCHD3	Coiled-coil-helix-coiled-coil-helix domain containing 3	0.488987548	0.03660934
AP2M1	Adaptor related protein complex 2 mu 1 subunit	0.487700783	0.048251772
TIPRL	TOR signaling pathway regulator	0.486363825	0.042595348
PRPS1	Phosphoribosyl pyrophosphate synthetase 1	0.46958207	0.04835945
PSMD2	Proteasome 26S subunit, non-ATPase 2	0.461959009	0.044919395
FARSB	Phenylalanyl-tRNA synthetase beta subunit	0.419564279	0.045741443

Supplementary Table 3. Significantly downregulated endocervical genes in the follicular vs. secretory phase

Symbol	Entrez Gene Name	logFC	Adj. *p* value
SERPINA5	Serpin family A member 5	-4.342656727	0.040952507
AL353803.2	RNA Gene	-3.936495969	0.038511708
ENPP3	Ectonucleotide pyrophosphatase/phosphodiesterase 3	-3.272466579	0.037507157
TUBB8P7	Tubulin beta 8 class VIII pseudogene 7	-2.715093098	0.046953498
ADH6	Alcohol dehydrogenase 6 (class V)	-2.510878053	0.043033761
AC008763.1	RNA Gene	-2.402216384	0.046287061
DNAH5	Dynein axonemal heavy chain 5	-1.94158518	0.046287061
TDRD6	Tudor domain containing 6	-1.848414252	0.03660934
TESMIN	Testis expressed metallothionein like protein	-1.842357862	0.046953498
AC092164.1	Uncharacterized	-1.793984353	0.038727705
LOC101929234	Uncharacterized	-1.784337003	0.044919395
NAPSA	Napsin A aspartic peptidase	-1.775433998	0.046953498
GOLGA2P5	Golgin A2 pseudogene 5	-1.718914126	0.046287061
ZNF233	Zinc finger protein 233	-1.712897961	0.034367731
CABYR	Calcium binding tyrosine phosphorylation regulated	-1.679896104	0.044919395
C6orf163	Chromosome 6 open reading frame 163	-1.639797528	0.04835945
AC012123.1	Uncharacterized RNA gene	-1.539812811	0.04835945
AC074212.1	Uncharacterized RNA gene	-1.53118335	0.034367731
AL355987.4	RNA Gene	-1.523194859	0.03660934
LINC01341	Long intergenic non-protein coding RNA 1341	-1.503947734	0.044919395
AC010487.1	Uncategorized	-1.455989461	0.038511708
AP006621.2	RNA Gene	-1.437475583	0.046287061
LRRC66	Leucine rich repeat containing 66	-1.409954097	0.044919395
PNPLA7	Patatin like phospholipase domain containing 7	-1.403469844	0.044919395
TSPAN10	Tetraspanin 10	-1.350207395	0.04835945
AC018755.1	Uncharacterized	-1.335347118	0.048251772
AVIL	Advillin	-1.279630769	0.044919395
ZNF737	Zinc finger protein 737	-1.251098502	0.03660934
TMEM9B-AS1	TMEM9B antisense RNA 1	-1.244342679	0.03660934
AC120053.1	Uncategorized	-1.221711179	0.038977475
SLC25A29	Solute carrier family 25 member 29	-1.210797498	0.044919395
DM1-AS	RNA Gene	-1.144758386	0.04835945
KIAA1107	KIAA1107	-1.139983849	0.03660934
ZNF391	Zinc finger protein 391	-1.124362736	0.044919395
ADHFE1	Alcohol dehydrogenase, iron containing 1	-1.111159388	0.037068701
FAM161A	Family with sequence similarity 161 member A	-1.110737182	0.03660934
PLA2G6	Phospholipase A2 group VI	-1.106518124	0.037507157
ZNF540	Zinc finger protein 540	-1.084497353	0.04293061
FAM13A	Family with sequence similarity 13 member A	-1.068612045	0.031708253
ACVR2B	Activin A receptor type 2B	-1.066242367	0.037507157
AC135178.1	Uncharacterized	-1.02591765	0.046287061
ZNF19	Zinc finger protein 19	-1.020717956	0.043033761
TFAP4	Transcription factor AP-4	-1.00452805	0.048251772
LINC01011	Long intergenic non-protein coding RNA 1011	-0.996631224	0.037507157
ZNF137P	Zinc finger protein 137, pseudogene	-0.983726767	0.038708533
RP4-714D9.5	Uncharacterized	-0.980762005	0.045931911
LOC100287896	Uncharacterized	-0.973107089	0.044919395
ZNF204P	Zinc finger protein 204, pseudogene	-0.971971653	0.044555005
ZNF503	Zinc finger protein 503	-0.970719704	0.046287061
L3MBTL1	Histone methyl lysine binding protein	-0.966126529	0.048251772

WBP1	WW domain binding protein 1	-0.943488609	0.044919395
ZNF285	Zinc finger protein 285	-0.94136829	0.037507157
DCST2	DC-STAMP domain containing 2	-0.939717497	0.046954048
FAM229A	Family with sequence similarity 229 member A	-0.936186525	0.043033761
ZNF681	Zinc finger protein 681	-0.935642393	0.04835945
GTF2IP20	General transcription factor IIi pseudogene 20	-0.930381232	0.044919395
AP002807.1	Uncategorized	-0.924449154	0.046287061
SRP14-AS1	SRP14 antisense RNA1 (head to head)	-0.91548326	0.046953498
HEXDC	Hexosaminidase D	-0.897765341	0.03660934
TSPOAP1	TSPO associated protein 1	-0.894120572	0.03660934
ZNF546	Zinc finger protein 546	-0.888506723	0.046287061
ZNF90	Zinc finger protein 90	-0.879251481	0.048251772
LOC171391	Uncharacterized	-0.85412727	0.038708533
VSIG10	V-set and immunoglobulin domain containing 10	-0.846210611	0.03660934
TDRP	Testis development related protein	-0.84513062	0.044919395
C19orf44	Chromosome 19 open reading frame 44	-0.833601379	0.044919395
EIF3J-AS1	EIF3J antisense RNA 1 (head to head)	-0.830398029	0.044919395
HSF4	Heat shock transcription factor 4	-0.825438943	0.046287061
CTC-462L7.1	Uncharacterized	-0.816893398	0.046287061
MTERF2	Mitochondrial transcription termination factor 2	-0.809987357	0.049805429
ECHDC2	Enoyl-CoA hydratase domain containing 2	-0.794332075	0.04835945
ZNF577	Zinc finger protein 577	-0.780862899	0.048251772
COQ8A	Coenzyme Q8A	-0.755824236	0.037507157
TTC28-AS1	TTC28 antisense RNA 1	-0.747189387	0.044919395
ZNF836	Zinc finger protein 836	-0.7454417	0.04835945
POLG2	DNA polymerase gamma 2, accessory subunit	-0.743592326	0.04835945
ZNF862	zinc finger protein 862	-0.73802097	0.046287061
ING5	Inhibitor of growth family member 5	-0.733293949	0.04835945
ZFP62	ZFP62 zinc finger protein	-0.73322482	0.03660934
ZNF626	Zinc finger protein 626	-0.720623533	0.03660934
KLHL24	Kelch like family member 24	-0.720249374	0.031708253
ZMYM3	Zinc finger MYM-type containing 3	-0.719690003	0.046287061
PAN2	PAN2 poly(A) specific ribonuclease subunit	-0.717326995	0.040375139
PPP1R3E	Protein phosphatase 1 regulatory subunit 3E	-0.715770587	0.044919395
TMCO6	Transmembrane and coiled-coil domains 6	-0.713230941	0.04835945
PEX6	Peroxisomal biogenesis factor 6	-0.708754978	0.048251772
PPFIBP2	PPFIA binding protein 2	-0.645403935	0.046332656
CIRBP	Cold inducible RNA binding protein	-0.629608116	0.04835945
APBB3	Amyloid beta precursor protein binding family B member 3	-0.604480725	0.03660934
KLHDC2	Kelch domain containing 2	-0.601946492	0.038511708
ASAP3	ArfGAP with SH3 domain, ankyrin repeat and PH domain 3	-0.597164422	0.034367731
SCAND2P	SCAN domain containing 2 pseudogene	-0.593935889	0.046287061
ZNF91	Zinc finger protein 91	-0.593918805	0.038511708
ANKRA2	Ankyrin repeat family A member 2	-0.592510184	0.03660934
KANSL1L	KAT8 regulatory NSL complex subunit 1 like	-0.586194649	0.049108032
FAN1	FANCD2 and FANCI associated nuclease 1	-0.577011256	0.037507157
PCMTD2	Protein-L-isoaspartate (D-aspartate) O-methyltransferase domain containing 2	-0.558134372	0.03660934
ZNF254	Zinc finger protein 254	-0.506892531	0.04835945
SIRT3	Sirtuin 3	-0.503616389	0.044919395
FAM214A	Family with sequence similarity 214 member A	-0.492915513	0.04835945

Supplementary Table 4. Canonical pathways associated with the follicular vs. secretory phase of endocervix

Ingenuity Canonical Pathways	-log(*p* value)	Ratio
Remodeling of Epithelial Adherens Junctions	7.25	0.116
Epithelial Adherens Junction Signaling	5.72	0.0616
Protein Ubiquitination Pathway	3.67	0.034
Germ Cell-Sertoli Cell Junction Signaling	3.42	0.0405
Regulation of Actin-based Motility by Rho	3.19	0.0556
Rapoport-Luebering Glycolytic Shunt	3.07	0.333
Phagosome Maturation	3	0.0405
Sertoli Cell-Sertoli Cell Junction Signaling	2.6	0.0337
RhoA Signaling	2.57	0.0403
tRNA Charging	2.48	0.0769
14-3-3-mediated Signaling	2.47	0.0382
Clathrin-mediated Endocytosis Signaling	2.28	0.029
Fcγ Receptor-mediated Phagocytosis in Macrophages and Monocytes	2.24	0.043
Axonal Guidance Signaling	2.08	0.0199
Cdc42 Signaling	2.03	0.0299
RhoGDI Signaling	1.93	0.0282
Signaling by Rho Family GTPases	1.88	0.0238
Guanine and Guanosine Salvage I	1.82	0.5
Spermidine Biosynthesis I	1.82	0.5
Putrescine Biosynthesis III	1.82	0.5
Gap Junction Signaling	1.76	0.0256
ILK Signaling	1.75	0.0254
Breast Cancer Regulation by Stathmin1	1.68	0.0244
TWEAK Signaling	1.53	0.0571
PRPP Biosynthesis I	1.52	0.25
Actin Cytoskeleton Signaling	1.51	0.022
Ethanol Degradation II	1.49	0.0541
Noradrenaline and Adrenaline Degradation	1.43	0.05
Role of PKR in Interferon Induction and Antiviral Response	1.41	0.0488
Tight Junction Signaling	1.4	0.024
PAK Signaling	1.37	0.0297
Pyrimidine Ribonucleotides Interconversion	1.33	0.0444

Supplementary Fig. 1

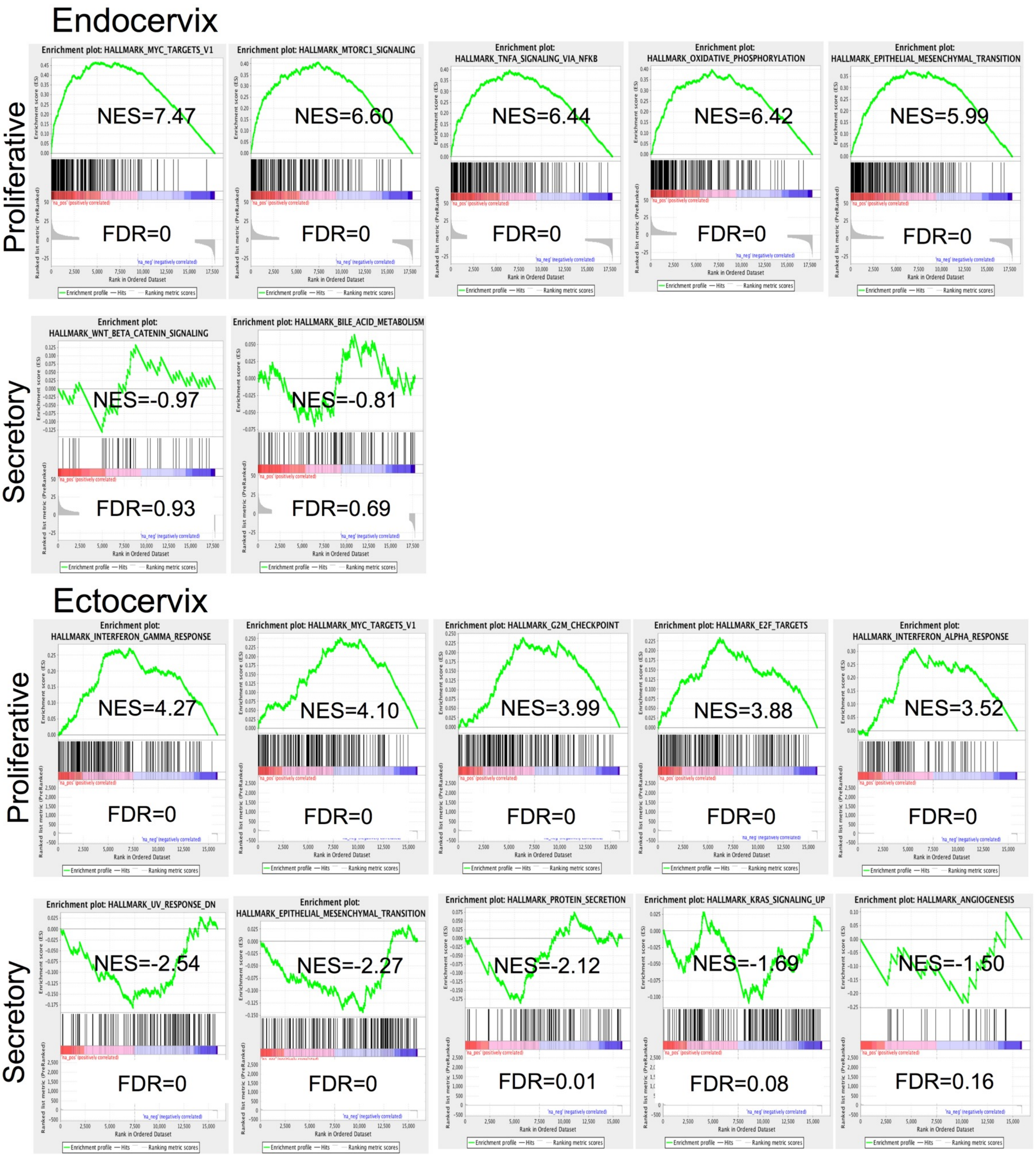

Suppl Fig. 1. GSEA using Hallmark gene sets. Top GSEA plots for gene sets which positively (+ normalized enrichment score (NES)) or negatively (-NES) correlated with a particular phase of the cycle in the endocervix and ectocervix. On the x-axis are genes ranked according to their expression from upregulated on the left to the downregulated genes on the right. The cumulative value of the enrichment score (y-axis) is represented by the green line. FDR is the false discovery rate.

Supplementary Fig. 2

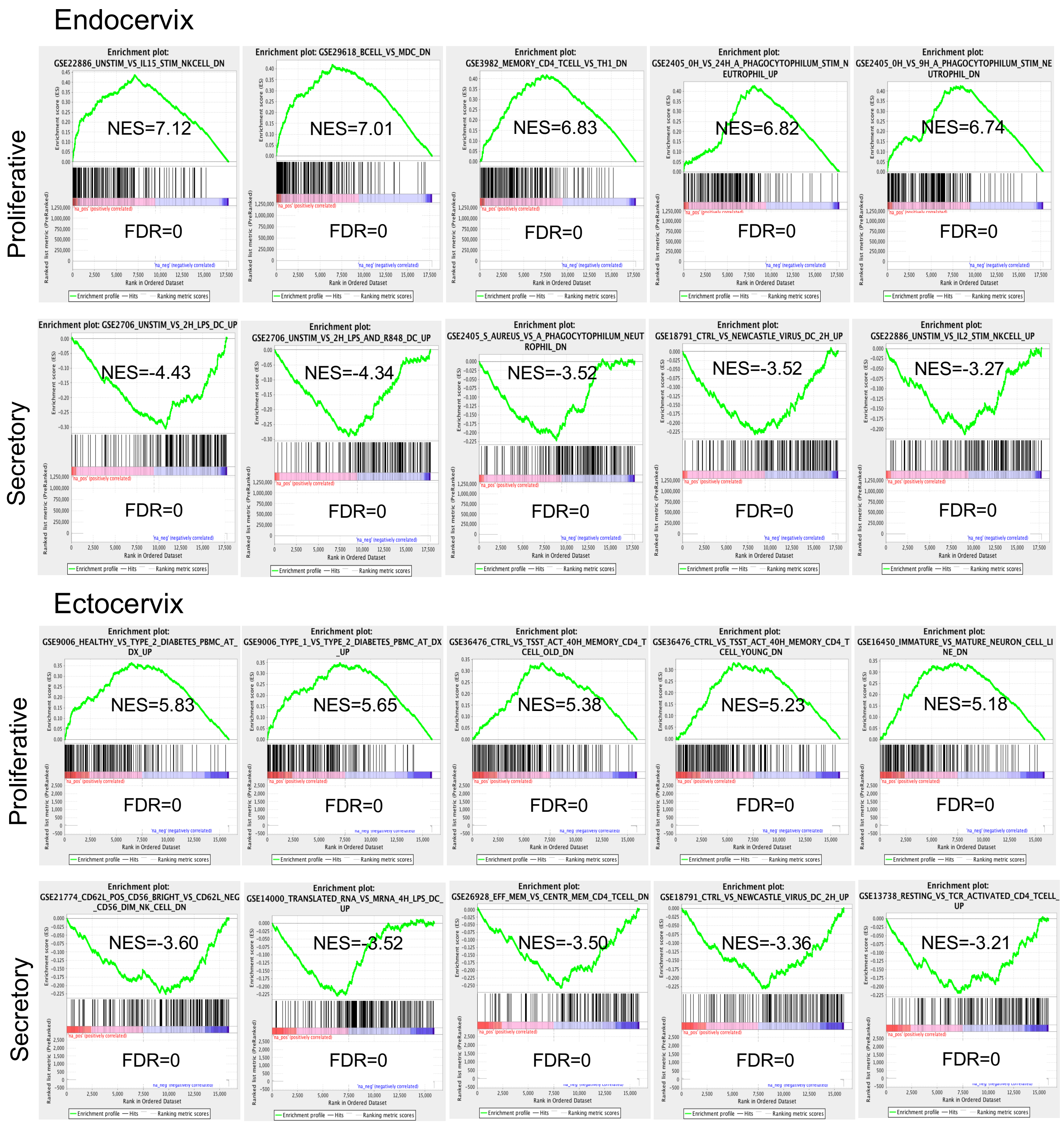

Suppl Fig. 2. GSEA using Immunologic Signatures gene sets. Top GSEA plots for gene sets which positively (+ normalized enrichment score (NES)) or negatively (- NES) correlated with a particular phase of the cycle in the endocervix and ectocervix. On the x-axis are genes ranked according to their expression from upregulated on the left to the downregulated genes on the right. The cumulative value of the enrichment score (y-axis) is represented by the green line. FDR is the false discovery rate.
